# Meta-Analysis for Clinical Evaluation of Xingnaojing Injection for the Treatment of Cerebral Infarction

**DOI:** 10.3389/fphar.2017.00485

**Published:** 2017-08-29

**Authors:** Xiao Ma, Yu X. Yang, Nian Chen, Qian Xie, Tao Wang, Xuan He, Jian Wang

**Affiliations:** ^1^College of Pharmacy, Chengdu University of Traditional Chinese Medicine Chengdu, China; ^2^Department of Pharmacy, Xindu District Shibantan Public Hospital Chengdu, China

**Keywords:** Xingnaojing injection, cerebral infarction, meta-analysis, systematic review, clinical evaluation

## Abstract

**Objective:** Xingnaojing injection (XNJ) is derived from An-Gong-Niu-Huang pill, a well-known traditional Chinese patent medicine, which is widely used for stroke. To evaluate the therapeutic effect of XNJ on cerebral infarction, an extensive meta-analysis was used.

**Methods:** Six major electronic databases including the Chinese Biomedical Database (CBM), Wanfang, the VIP medicine information system (VMIS) and the China National Knowledge Infrastructure (CNKI), PubMed, Embase, and the Cochrane Library were examined to retrieve randomized controlled trials designed to evaluate the clinical efficacy of XNJ in treating CI before November 26, 2016.

**Results:** There were 53 randomized controlled trials with 4915 participants in this study. The results reflected that compared with the conventional therapy (CT) alone, XNJ could significantly improve the overall response rate (*OR* = 3.56, 95% CI [2.94, 4.32], *P* < 0.00001), and clinical symptom (including increasing activities of daily living (ADL, *MD* = 10.23, 95% CI [9.47, 10.99], *P* < 0.00001), and reduce infarction size (*MD* = -1.83, 95% CI [-2.49, -1.16], *P* < 0.00001)). However, there was no significant difference between the XNJ treatment and conventional therapy in Glasgow Coma Scale (GCS, *P* = 0.32). Neurological deficit score demonstrated that XNJ could significantly reduce the score in two different evaluation criterions as National Institutes of Health Stroke Scale (NIHSS, *MD* = -3.44, 95% CI [-4.52, -2.36], *P* < 0.00001), and the Chinese Stroke Scale (CSS, *MD* = -5.72, 95% CI [-6.94, -4.50], *P* < 0.00001). Additionally, serum MMPs, including MMP-2 and MMP-9 were significantly reduced by XNJ treatment compared with conventional therapy (*MD* = -11.24, 95% CI [-20.83, -1.65], *P* = 0.02; *MD* = -25.08, 95% CI [-35.49, -14.67], *P* < 0.00001, respectively). Moreover, XNJ was able to improve hemorrheology in reducing whole blood viscosity, plasma viscosity, and hematocrit (*MD* = -1.44, 95% CI [-2.18, 0.70], *P* = 0.001; *MD* = -0.22, 95% CI [-0.37, -0.07], *P* = 0.003; *MD* = -3.63, 95% CI [-6.23, -1.03], *P* = 0.006, respectively). The therapeutic efficacy of XNJ was found associated with improving hemodynamics (increasing peak-flow rate, and average velocity) (*MD* = 12.66, 95% CI [10.50, 14.81], *P* < 0.00001; *MD* = 9.90, 95% CI [8.63, 11.17], *P* < 0.00001). XNJ was also related to reducing cholesterol and triglyceride (*MD* = -1.06, 95% CI [-1.21, -0.92], *P* < 0.00001; *MD* = -1.05, 95% CI [-1.12, -0.97], *P* < 0.00001).

**Conclusion:** Despite the sample size and the poor quality of the included studies of this review, the results of the research showed that XNJ might be a beneficial therapeutic method for the treatment of cerebral infarction.

## Introduction

Stroke is the one of the most common diseases worldwide, with a high disability, mortality and recurrence rate, and usually leads to serious damage of central nervous system (Johnston et al., 2009). There are three types of stroke, including ischemia stroke, cerebral hemorrhage, and cerebral thrombosis. Ischemia stroke, also been known as cerebral infarction, is the most commonly seen in stroke, with the highest morbidity about 70% (Luo, 2010). Although the main pathology clearly defined, namely that brain tissue hypoxic ischemia is caused by atherosclerosis of cerebral arteries, alone or with superimposed thrombosis, hypertension, diabetes mellitus, and cardiovascular disease (Zhou, 2010; Hata et al., 2011), the corresponding precautionary measures are still limited in preventing the incidence rate.

Currently, the conventional therapy (CT), including thrombolysis, restoring blood supply to ischemic area, controlling cerebral edema, cerebral protection agents, preventing and treating complications, controlling hypertension, reducing blood viscosity, and so on, is the main clinical therapy for cerebral infarction (Deng et al., 2011). Recently, the theory that complementary medicine can substantial improve the disease has been put forward (Sze et al., 2005).

Xingnaojing injection (XNJ) is composed of musk, synthetic borneol, *Curcuma aromatica* Salisb, and *Gardenia jasminoides* J.Ellis derived from a classic traditional Chinese emergency prescription named An-Gong-Niu-Huang pill. It is widely used to treat nervous system disorders in China (Guo et al., 2014). Numerous studies indicating that the main active ingredients are muscone, borneol, camphor, curcumin, and curzerenone, and demonstrated borneol could be as a quality control substance, and it should be no less than 0.7 mg per ml XNJ injection. According to the above, the clinical widely used of XNJ are the quality assurance. In recent years, there was an increase of clinical and pharmacological research indicating that XNJ can ameliorate brain function and promote the recovery of consciousness. The mechanisms might include scavenging free radicals, improving cerebral edema and hypoxia, and increasing the metabolic rate and activity of brain cells (He et al., 2006, 2007; Guo, 2013; Ma et al., 2014). However, there is no comprehensive and systematic evidence to confirm its clinical efficacy. Therefore, a comprehensive meta-analysis was performed in order to systematically evaluate the effectiveness of XNJ combined with CT for the treatment of cerebral infarction compared with the CT alone.

## Materials and Methods

### The Literature Search Strategy

All RCTs concerning the effectiveness and safety of XNJ in treating cerebral infarction were retrieved from six databases, including PubMed, EMBASE, Cochrane Library, Wanfang database, VIP medicine information system, and China National Knowledge Infrastructure Database (CNKI) from inception to November 26, 2016, in terms of the keywords of “Xingnaojing injection” [Title/Abstract] AND “ischemia stroke” [Title/Abstract] OR “cerebral infarction” [Title/Abstract]. The searched results were downloaded for the further screening.

### Inclusion and Excluded Criteria

The literatures screening was performed by two investigators (Yuxue Yang and Tao Wang) independently. The title, abstract, and full-text were browsed in sequence to evaluate whether the study should be included according to the inclusion and exclusion criteria. The screening criteria were predesigned before screening, and the discussion was organized to evaluate the appropriateness of the studies if any divergences were observed.

#### Inclusion Criteria

The inclusion criteria were as follows: (1) Randomized controlled trials (RCTs); (2) Patients who were suffering from cerebral infarction according specific diagnosis criteria; (3) There were no other medicines in combination with the CT in the experimental group, except for Xingnaojing injection, compared with the CT as a control; (4) Treatment duration had to be at least 14 days; (5) One or more outcome measures, including the overall response rate, neurological deficit score, serum level of matrix metalloproteinase (MMPs), hemorheology, blood lipid, hemodynamic, clinical symptom improvement (including activities of daily living, ADL; Glasgow Coma Scale, GCS; and infarction size) a must be included in each study.

#### Exclusion Criteria

The study met with the following items would be excluded: (1) Duplicated articles, reviews, non-clinical studies, case observations; (2) Trials were not RCTs; (3) Combined with any other specific medicines in control group or experimental during the treatment.

### Data Extraction and Risk of Bias Assessment

Two investigators (Yuxue Yang and Tao Wang) independently performed the data extraction and quality assessment of the included studies. The baseline information, including first author, publication year, the cases of experimental and control groups, respectively, interventions of experimental group, outcome measures were extracted to yield a conclusion table.

All the included RCTs were assessed for methodological quality using the Cochrane Handbook for Systematic Reviews of Interventions (Higgins et al., 2003). Five items, including random sequence generation, allocation concealment, blinding of participants and personnel, blinding of outcome assessment, incomplete outcome data and selective reporting were used for the methodological quality of each included studies. The quality of each item was assessed using the three levels of “low risk,” “high risk,” or “unclear risk.”

### Data Analysis

Cochrane Review Manager 5.3 (Cochrane Collaboration) was used for statistical analysis. Dichotomous variables, such as overall response rate, were presented as odds ratio (*OR*), while the continuous variables, including the neurological deficit score, serum level of matrix metalloproteinase (MMPs), hemorheology, blood lipid, hemodynamic were presented as mean difference (*MD*) with 95% confidence intervals (95% CI). Additionally, the statistical heterogeneity was estimated using *P*-value and *I*-square (*I*^2^) statistic tests. Data with *P* ≥ 0.10 and *I*^2^ ≤ 50% were defined as low heterogeneity, and a fixed-effects model would be used for the meta-analysis, whereas, a random-effects model was used for data with substantial heterogeneity (*P* < 0.10, *I*^2^> 50%). The publication bias was estimated by a funnel plot.

## Results

### Search Results

**FIGURE 1:**
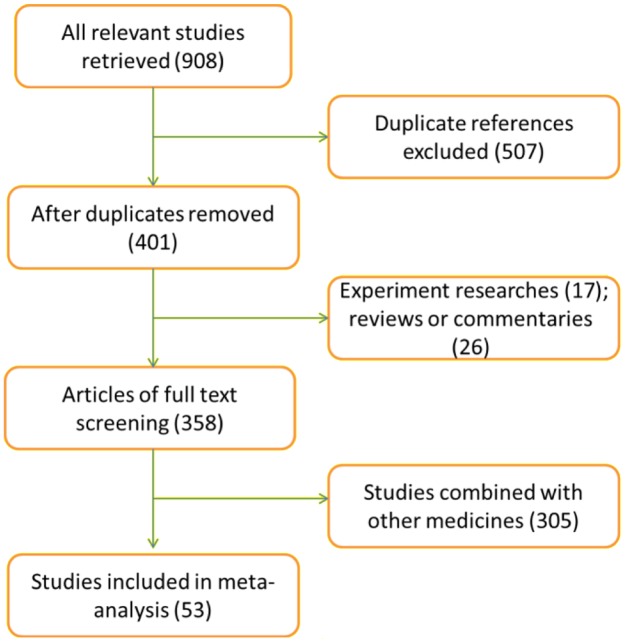
Flow diagram of the literature screening.

Nine hundred and eight publications were retrieved according to the search strategy. After the title and abstract browsing, 507 articles were removed for duplicated. 348 reviews were excluded for the following reasons: non-randomized controlled studies, animal studies, ambiguous outcome measures and overlapping data with another study, and combination with other medicines. Ultimately, 53 eligible articles with 4915 participants (2475 cases in the experimental group, 2440 cases in the control group) were included in the meta-analysis according to the inclusion and exclusion criteria after the full-text reading (**Figure 1**).

### Study Characteristics and Methodological Quality of Included Trials

Among the 53 included trails, which were published between 1999 and 2016, all were RCTs with a comparison between a combination of XNJ and CT with CT treatment alone. The dosage of administered XNJ ranged from 10 to 40 mL/day via intravenous drip. There was no significant difference between the experimental and control groups in general information (**Table 1**).

**Table 1:** Characteristics and outcome measures of included studies.

Included research (year)	E/C	Intervening measure (E/C)	Dosage of XNJ	Duration	Outcome measures
Wu et al., 1999	20/20	XNJ+ CT vs. CT	40 mL/day	15 days	Overall response rate
Chen, 2006	39/30	XNJ+ CT vs. CT	20 mL/day	20 days	Overall response rate, Hemorheology, Blood lipid
Zhang et al., 2006	32/28	XNJ+ CT vs. CT	30 mL/day	15 days	Neurological deficit score
Li et al., 2008	27/27	XNJ+ CT vs. CT	20 mL/day	2 weeks	GCS
Su, 2008	39/30	XNJ+ CT vs. CT	20 mL/day	20 days	Overall response rate
Wu, 2008	40/40	XNJ+ CT vs. CT	20 mL/day	2 weeks	Overall response rate
Chen and Wang, 2009	73/84	XNJ+ CT vs. CT	30 mL/day	2 weeks	Neurological deficit score
Lu and Zhang, 2009	76/64	XNJ+ CT vs. CT	40 mL/day	15 days	Neurological deficit score
Qiu, 2009	30/30	XNJ+ CT vs. CT	20 mL/day	3 weeks	Hemorheology, Overall response rate, Blood lipid
Wu, 2009	54/52	XNJ+ CT vs. CT	20 mL/day	2 weeks	Overall response rate, neurological deficit score
Jiang, 2010	30/28	XNJ+ CT vs. CT	20 mL/day	15 days	Overall response rate
Lian and Dong, 2010	50/50	XNJ+ CT vs. CT	30 mL/day	2 weeks	Overall response rate, neurological deficit score
Chen, 2011	30/30	XNJ+ CT vs. CT	20 mL/day	2 weeks	Overall response rate, neurological deficit score
Han et al., 2011	43/43	XNJ+ CT vs. CT	20 mL/day	20–28 days	Neurological deficit score, Overall response rate
Li and Hou, 2011	30/30	XNJ+ CT vs. CT	20 mL/day	2 weeks	Neurological deficit score, Overall response rate
Qin et al., 2011	40/40	XNJ+ CT vs. CT	20–30 mL/day	2 weeks	Overall response rate, neurological deficit score
Tong and Zhu, 2011	70/66	XNJ+ CT vs. CT	30 mL/day	2 weeks	Overall response rate, neurological deficit score
Wang, 2011	30/30	XNJ+ CT vs. CT	20 mL/day	3 weeks	Hemorheology, Blood lipid, neurological deficit score
Wang, 2011	40/40	XNJ+ CT vs. CT	20 mL/day	2 weeks	Overall response rate, neurological deficit score
Yang, 2011	62/62	XNJ+ CT vs. CT	20 mL/day	15 days	Overall response rate
Zang et al., 2011	49/49	XNJ+ CT vs. CT	20 mL/day	2 weeks	Overall response rate
Li et al., 2012	43/43	XNJ+ CT vs. CT	20 mL/day	2 weeks	Neurological deficit score, Overall response rate
Li et al., 2012	30/31	XNJ+ CT vs. CT	20 mL/day	2 weeks	Neurological deficit score, Overall response rate, Blood lipid
Tong and Zhu, 2012	64/66	XNJ+ CT vs. CT	30 mL/day	2 weeks	Overall response rate, neurological deficit score
Wang, 2012	100/100	XNJ+ CT vs. CT	20 mL/day	30 days	NIHSS, Overall response rate
Dong and Fu, 2013	35/33	XNJ+ CT vs. CT	20 mL/day	2 weeks	Neurological deficit score
Lou, 2013	75/75	XNJ+ CT vs. CT	40 mL/day	2 weeks	Overall response rate, neurological deficit score
Qian and Jia, 2013	40/40	XNJ+ CT vs. CT	20 mL/day	2 weeks	Overall response rate, neurological deficit score
Zeng, 2013	49/49	XNJ+ CT vs. CT	20 mL/day	2 weeks	Overall response rate
Jia et al., 2014	96/96	XNJ+ CT vs. CT	20 mL/day	2 weeks	Infarction size, neurological deficit score
Luo, 2014	60/60	XNJ+ CT vs. CT	20 mL/day	2 weeks	Neurological deficit score, Overall response rate, ADL, Hemorheology
Lv, 2014	62/62	XNJ+ CT vs. CT	20 mL/day	15 days	Overall response rate, MMPs
Shen et al., 2014	45/41	XNJ+ CT vs. CT	20 mL/day	2 weeks	Overall response rate, GCS
Sun, 2014	38/38	XNJ+ CT vs. CT	20 mL/day	2 weeks	Overall response rate, MMPs
Wang et al., 2014	30/30	XNJ+ CT vs. CT	20–30 mL/day	2 weeks	Overall response rate, neurological deficit score
Wei and Shi, 2014	50/50	XNJ+ CT vs. CT	40 mL/day	1 week	NHISS
Yin et al., 2014	52/52	XNJ+ CT vs. CT	20 mL/day	2 weeks	Overall response rate, MMP-9
Yin and Wei, 2014	20/20	XNJ+ CT vs. CT	20 mL/day	2 weeks	Overall response rate, NHISS
Zhang, 2014	49/49	XNJ+ CT vs. CT	10 mL/day	30 days	Neurological deficit score
An et al., 2015	64/64	XNJ+ CT vs. CT	20 mL/day	2 weeks	Overall response rate, Infarction size, neurological deficit score
Liao et al., 2015	24/23	XNJ+ CT vs. CT	20 mL/day	NR	Overall response rate
Luo et al., 2015	50/50	XNJ+ CT vs. CT	10–20 mL/day	4 weeks	Overall response rate, Hemodynamics
Sun et al., 2015	42/42	XNJ+ CT vs. CT	20 mL/day	NR	MMP-9, Overall response rate
Wang and Huang, 2015	60/60	XNJ+ CT vs. CT	10–20 mL/day	20 days	Neurological deficit score, ADR
Zhao et al., 2015	35/35	XNJ+ CT vs. CT	20 mL/day	2 weeks	Overall response rate, neurological deficit score
Cai and Zhao, 2016	58/58	XNJ+ CT vs. CT	20 mL/d	2 weeks	MMPs, Overall response rate
Lin, 2016	30/30	XNJ+ CT vs. CT	20 mL/day	10 days	Neurological deficit score
Lu et al., 2016	35/35	XNJ+ CT vs. CT	30 mL/day	2 weeks	Overall response rate, neurological deficit score, Infarction size
Lu et al., 2016	35/35	XNJ+ CT vs. CT	30 mL/day	2 weeks	ADL
Wang and Wang, 2016	72/72	XNJ+ CT vs. CT	20 mL/day	30 days	Hemodynamics, Overall response rate
Wang and Wang, 2016	40/40	XNJ+ CT vs. CT	20 mL/day	2 weeks	Overall response rate
Xiong et al., 2016	48/48	XNJ+ CT vs. CT	20 mL/day	2 weeks	Overall response rate
Yang, 2016	40/40	XNJ+ CT vs. CT	20 mL/day	15 days	Overall response rate

*E, experimental group; C, control group; NR, no report*.

**FIGURE 2:**
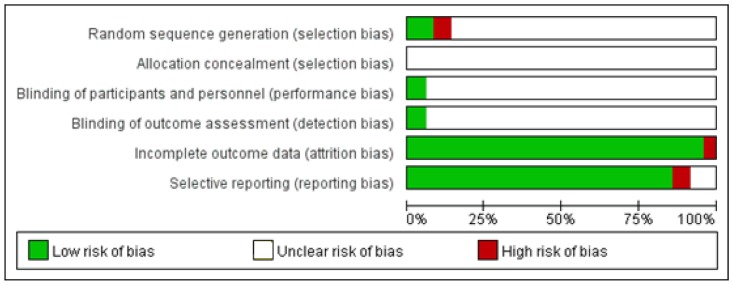
Methodological quality assessment of the risk of bias for each included study.

The methodological quality of included studies was estimated according to the Cochrane risk of bias estimation. All of the included trails mentioned randomized allocation, whereas, only 5 of them mentioned the appropriate generation of the random allocation sequence (Qin et al., 2011; Dong and Fu, 2013; Liao et al., 2015; Lu and Liu, 2016; Lu et al., 2016). There was no trails mentioned allocation concealment. Three of the 53 studies stated double blind design (blinding of participants and blinding of outcome assessment) (Li et al., 2008; Chen and Wang, 2009; Lv, 2014). Two of 53 studies were published with a high risk of incomplete outcome (Han et al., 2011; Wang and Jiang, 2016), and the risk of selective reporting was high for the three trials (Lian and Dong, 2010; Chen, 2011; Wang, 2012) (**Figure 2**).

### Analysis and Subgroup Analysis for Outcome Measures

#### The Overall Response Rate

**FIGURE 3:**
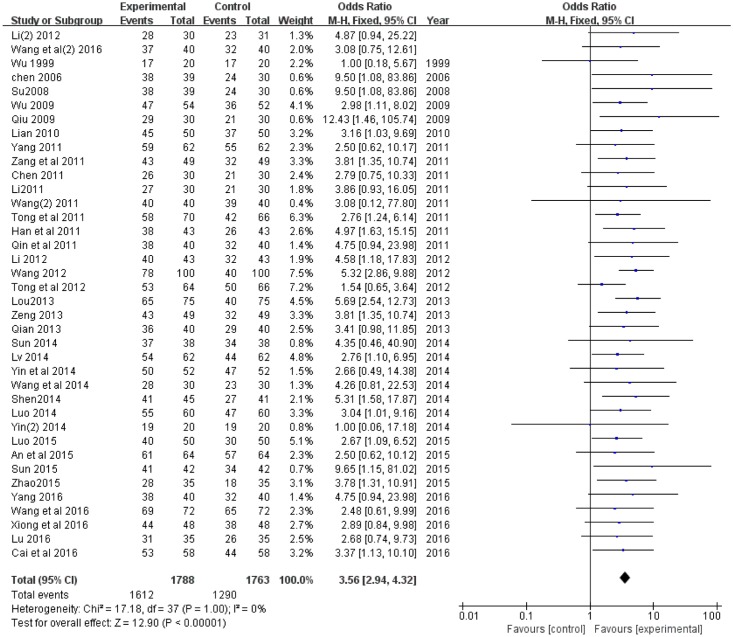
Forest plot of overall response rate comparing the experimental and control groups. *I*^2^ and *P* are the criterion for the heterogeneity test, 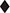 pooled odds ratio, 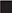 odds ratio and 95% CI.

Thirty-eight of the 53 trials (Wu et al., 1999; Chen, 2006, 2011; Su, 2008; Wu, 2008; Qiu, 2009; Lian and Dong, 2010; Han et al., 2011; Li and Hou, 2011; Qin et al., 2011; Tong and Zhu, 2011; Wang, 2011, 2012; Yang, 2011, 2016; Zang et al., 2011; Li and Su, 2012; Li et al., 2012; Tong and Zhu, 2012; Lou, 2013; Qian and Jia, 2013; Zeng, 2013; Luo, 2014; Lv, 2014; Shen et al., 2014; Sun, 2014; Wang et al., 2014; Yin and Wei, 2014; Yin et al., 2014; An et al., 2015; Luo et al., 2015; Sun et al., 2015; Zhao et al., 2015; Cai and Zhao, 2016; Lu et al., 2016; Wang and Jiang, 2016; Wang and Wang, 2016; Xiong et al., 2016) compared the overall response rate between XNJ combined with CT treatment and single CT treatment. There was no heterogeneity (*P* = 1.0, *I*^2^ = 0%), and a fixed-effect model was used to carry out the meta-analysis. An *OR* with 95% CI was used to present the comparison of overall response rate between the experimental and control groups (*OR* = 3.56, 95% CI [2.94, 4.32], *P* < 0.00001). It revealed that XNJ could significantly increase the treatment efficacy of CT for cerebral infarction (**Figure 3**).

#### The Neurological Deficit Score

**FIGURE 4:**
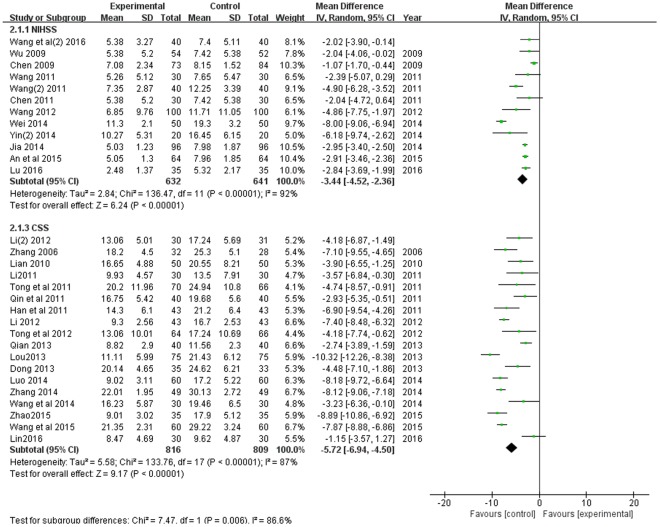
Forest plot of neurological deficit score comparing the experimental and control groups. *I*^2^ and *P* are the criterion for the heterogeneity test, 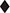 pooled odds ratio, 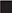 odds ratio and 95% CI.

Thirty trials with 3518 participants measured the neurological deficit score, and there were two evaluation criterions, including the National Institutes of Health Stroke Scale (NIHSS) (Chen and Wang, 2009; Wu, 2009; Chen, 2011; Wang et al., 2011; Wang, 2011, 2012; Jia et al., 2014; Wei and Shi, 2014; Yin et al., 2014; An et al., 2015; Lu et al., 2016; Wang and Wang, 2016), and the Chinese Stroke Scale (CSS, the fourth national cerebral vascular disease in 1995) (Zhang et al., 2006; Lian and Dong, 2010; Han et al., 2011; Li and Hou, 2011; Qin et al., 2011; Tong and Zhu, 2011; Li et al., 2012; Li and Su, 2012; Tong and Zhu, 2012; Dong and Fu, 2013; Lou, 2013; Qian and Jia, 2013; Luo, 2014; Wang et al., 2014; Zhang, 2014; Wang and Huang, 2015; Zhao et al., 2015; Lin, 2016) used. As shown in **Figure 4**, there were substantial heterogeneity both in the two subgroups (*P* < 0.00001, *I*^2^ = 92%; *P* < 0.00001, *I*^2^ = 87%). A random effects model was used to pool this meta-analysis. The result of this meta-analysis showed that the neurological deficit score of patients in the experimental group was much lower, compared with the control group according to both of the two evaluation criterions (*MD* = -3.44, 95% CI [-4.52, -2.36], *P* < 0.00001; *MD* = -5.72, 95% CI [-6.94, -4.50], *P* < 0.00001).

#### Serum Levels of MMPs

**FIGURE 5:**
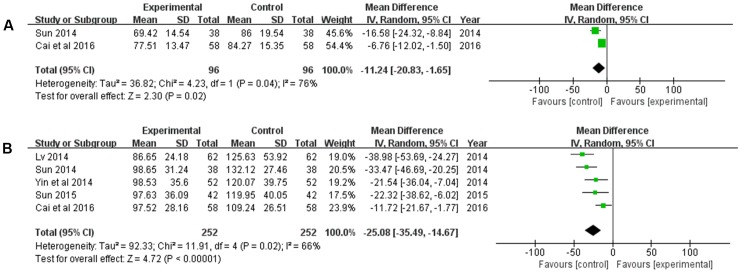
Forest plot of serum levels of MMPs comparing the experimental and control groups. **(A)** Forest plot of serum level of MMP-2; **(B)** forest plot of serum level of MMP-9. *I*^2^ and *P* are the criterion for the heterogeneity test, 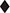 pooled odds ratio, 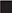 odds ratio and 95% CI.

The serum levels of MMP-2 and MMP-9 were measured. There were two studies (Sun, 2014; Cai and Zhao, 2016) involving 192 patients measured the level of MMP-2, and 5 studies with 504 participants measured the level of MMP-9. The random-effects model was used to pool this meta-analysis for the significant heterogeneity of them (*P* = 0.04, *I*^2^ = 76%; *P* = 0.02, *I*^2^ = 66%). The pooled analysis showed that compared with the CT, the XNJ could significantly reduce the serum levels of MMP-2, and MMP-9 (*MD* = -11.24, 95% CI [-20.83, -1.65], *P* = 0.02; *MD* = -25.08, 95% CI [-35.49, -14.67], *P* < 0.00001) (**Figure 5**).

#### Hemorrheology Improvement

**FIGURE 6:**
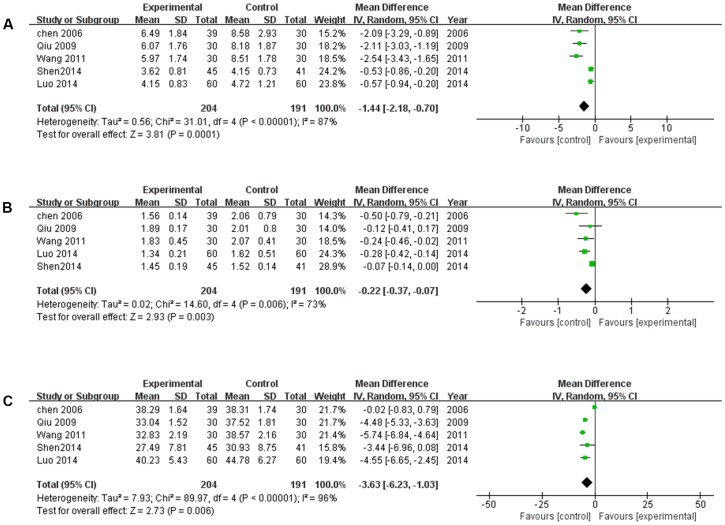
Forest plot of hemorrheology improvement comparing the experimental and control groups. **(A)** Forest plot of whole blood viscosity; **(B)** forest plot of plasma viscosity. **(C)** Forest plot of hematocrit. *I*^2^ and *P* are the criterion for the heterogeneity test, 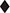 pooled odds ratio, 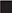 odds ratio and 95% CI.

The hemorrheology, including whole blood viscosity (WBV), plasma viscosity (PV), and hematocrit (HCT) was measured in five studies (Chen, 2006; Qiu, 2009; Wang et al., 2011; Luo, 2014; Shen et al., 2014). The random-effects model was used for the substantial heterogeneity of the three subgroups (*P* < 0.00001, *I*^2^ = 87%; *P* = 0.006, *I*^2^ = 73%; *P* < 0.00001, *I*^2^ = 96%). As shown in **Figure 6**, XNJ was more effective in improving hemorrheology by reducing WBV, PV, and HCT comparing with CT (*MD* = -1.44, 95% CI [-2.18, 0.70], *P* = 0.001; *MD* = -0.22, 95% CI [-0.37, -0.07], *P* = 0.003; *MD* = -3.63, 95% CI [-6.23, -1.03], *P* = 0.006).

#### Hemodynamics Improvement

**FIGURE 7:**
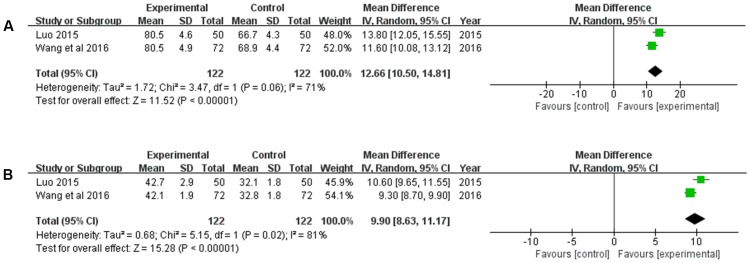
Forest plot of hemodynamics improvement comparing the experimental and control groups. **(A)** Forest plot of peak-flow rate; **(B)** forest plot of average velocity. *I*^2^ and *P* are the criterion for the heterogeneity test, 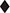 pooled odds ratio, 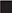 odds ratio and 95% CI.

There were two studies (Luo et al., 2015; Wang and Jiang, 2016) reporting the hemodynamics (peak-flow rate, and average velocity) improvement. Heterogeneity between the two studies was significant (*P* = 0.06, *I*^2^ = 71%; *P* = 0.02, *I*^2^ = 81%). Hence, a random-effects model was used to pool the meta-analysis. The results revealed that compared with the CT, XNJ could remarkably increase the peak-flow rate, and average velocity (*MD* = 12.66, 95% CI [10.50, 14.81], *P* < 0.00001; *MD* = 9.90, 95% CI [8.63, 11.17], *P* < 0.00001) (**Figure 7**).

#### Blood Lipid Amelioration

**FIGURE 8:**
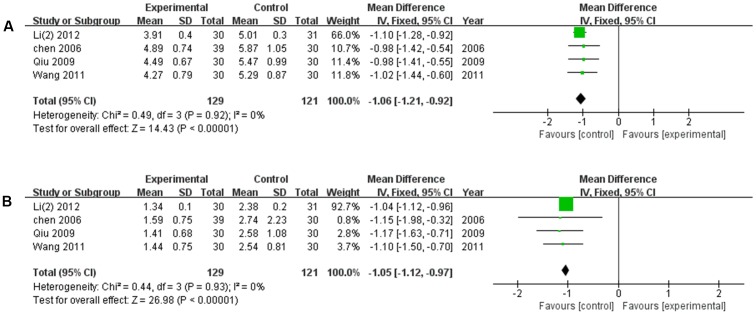
Forest plot of blood lipid amelioration comparing the experimental and control groups. **(A)** Forest plot of cholesterol; **(B)** forest plot of triglyceride. *I*^2^ and *P* are the criterion for the heterogeneity test, 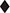 pooled odds ratio, 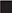 odds ratio and 95% CI.

There were four studies (Chen, 2006; Qiu, 2009; Wang et al., 2011; Li and Su, 2012) reported the amelioration of blood lipid after the treatment of XNJ and CT, respectively. There was no significant heterogeneity among the individual trails (*P* = 0.92, *I*^2^ = 0%; *P* = 0.93, *I*^2^ = 0%). The meta-analysis, using the fixed-effects model, revealed that compared with the CT, XNJ could significantly reduce the levels of cholesterol and triglyceride in blood (*MD* = -1.06, 95% CI [-1.21, -0.92], *P* < 0.00001; *MD* = -1.05, 95% CI [-1.12, -0.97], *P* < 0.00001) (**Figure 8**).

#### Clinical Symptoms Improvement

**FIGURE 9:**
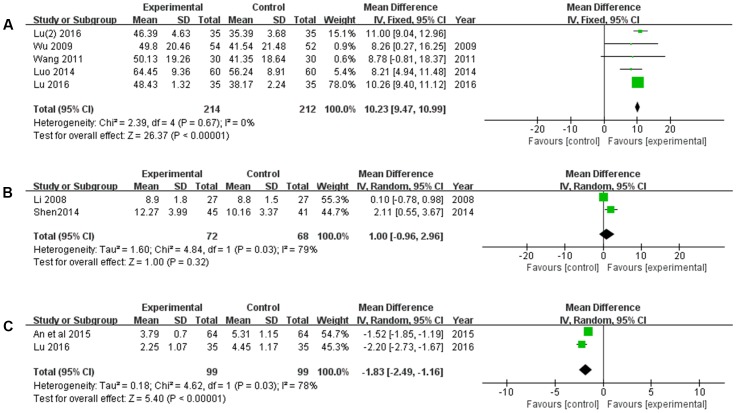
Forest plot of clinical symptom improvement comparing the experimental and control groups. **(A)** Forest plot of activities of daily living; **(B)** forest plot of Glasgow Coma Scale; **(C)** forest plot of infarct size. *I*^2^ and *P* are the criterion for the heterogeneity test, 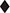 pooled odds ratio, 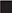 odds ratio and 95% CI.

The clinical symptoms, including ADL, GCS, and infarct size were measured. There were five trails (Wu et al., 1999; Wang et al., 2011; Luo, 2014; Lu et al., 2016; Lu and Liu, 2016) reported the ADL improvement. As shown in **Figure 9A**, XNJ could significantly improve the ADL of patients with cerebral infarction, compared with the CT (*MD* = 10.23, 95% CI [9.47, 10.99], *P* < 0.00001). There was no significant heterogeneity among the individual studies (*P* = 0.67, *I*^2^ = 0%), and a fixed-effects model was used to pool this meta-analysis. As for coma scale improvement, there was no significant difference between the experimental and control groups (*MD* = 1.00, 95% CI [-0.96, 2.96], *P* = 0.32) (**Figure 9B**). Additionally, XNJ could significantly decrease the infarct size (*MD* = -1.83, 95% CI [-2.49, -1.16], *P* < 0.00001). Heterogeneity between the studies was substantial, and a random-effects model was used (*P* = 0.03, *I*^2^ = 78%) (**Figure 9C**).

#### Publication Bias

**FIGURE 10:**
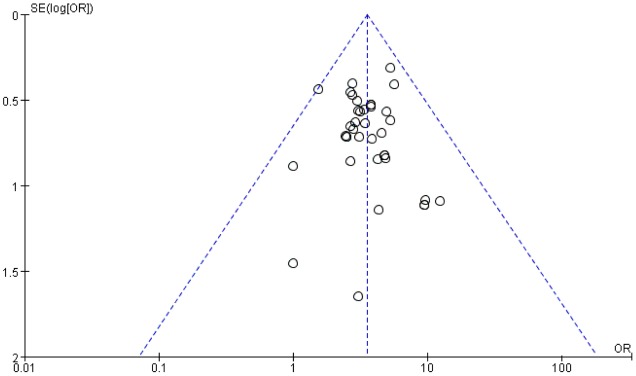
Funnel plot for the publication bias of the overall response rate.

A funnel plot was used to evaluate the publication bias. A total of 38 trails included in the funnel plot of the overall response rate. As shown in **Figure 10**, there was no significant asymmetry observed.

## Discussion

Cerebral infarction, known as ischemia stroke, is one of the common cerebrovascular diseases with high morbidity and mortality and seriously endangering the health and daily life of patients (Broussalis et al., 2011). It has been widely recognized one of the primary causes of death and disability both in developed and developing countries (Broussalis et al., 2011). CT, including thrombolysis, improving microcirculation, the applying of neuroprotective agents restoring, blood supply to ischemic area, controlling cerebral edema, preventing and treating complications, controlling hypertension, reducing blood viscosity, etc. is the main clinical therapy for the patients with cerebral infarction in current years. However, the motor weakness on one or both sides of body may be caused by the frequently used of conventional agents (Li et al., 2014). Therefore, the more effective agents for cerebral infarction patients are desirable.

Traditional Chinese medicine has been used to treat stroke in China during the past 2,000 years. Advanced pharmaceutical technologies have led to the development of many oral agents and injections for the prevention and treatment of stroke that are based on well-known traditional Chinese medical prescriptions (Wu et al., 2007). It has been demonstrated that XNJ to be an effective agent for reducing brain injuries, and enhancing functional recovery. However, a comprehensive and systematic evaluation of XNJ for the treatment of cerebral infarction is rare, according to current rigorous international standards (He et al., 2012). The aim of this review was to provide an internationally accessible systematic review of the clinical efficacy and safety of XNJ for the cerebral infarction.

A recent meta-analysis indicated that XNJ could significantly increase efficacy rate, decrease neurological deficit scores and the serum level of TNF-α of the patients of stroke. The statistical analysis found that the primary mechanism of XNJ activity was neuroprotective effect via improvement of cerebral circulation and blood flow and a reduction of cerebral edema, ferritin, and inflammation (Peng et al., 2014). In this review, we further explored the effect of XNJ on cerebral infarction and provided extended findings. First of all, overall response rate and neurological deficit score were chosen to measure the clinical efficacy of XNJ according to the “the criteria for the degree of defect of the clinical function of the patients in stroke” in the fourth academic conference on cerebrovascular disease (The Fourth National Academic Conference on Cerebro-vascular Diseases, 1996), which were direct correlated to improvement of patients with cerebral infarction. Compared with CT alone, XNJ combined with CT was associated with relatively higher overall response rate and lower neurological deficit score (*P* < 0.00001, *P* < 0.00001, respectively). The combination therapy also alleviated the clinical symptom of patients with cerebral infarction, including improving ADL, reducing infarction size (*P* < 0.00001, *P* < 0.00001, respectively). It did not result in a statistically significant improvement of the GCS (*P* = 0.32). The result of GCS improvement was based on two small-samples. Therefore, additional trails are necessary for a better data basis.

Meanwhile, the mechanism of XNJ activity for cerebral infarction was explored. The accumulating data revealed that MMPs are deleterious in stroke, in particular MMP-2 and MMP-9 (Rosenberg, 2002; Fatar et al., 2005). The result of our meta-analysis suggested that based on the CT, XNJ could significantly lower the level of MMP-2 and MMP-9 (*P* = 0.02, *P* < 0.00001). Additionally, the hermorrheology (including WBV, PV, and HCT) and hemodynamics (Vp, and Vm) were measured to evaluate the treatment effect of XNJ on cerebral infarction. As shown in **Figure 6**, XNJ could significantly improve the hemorrheology by reducing WBV, PV, and HCT (*P* = 0.0001, *P* = 0.003, *P* = 0.006, respectively) based on the CT. And at the same time, the hemodynamics was also significantly improved (*P* < 0.00001, *P* < 0.00001). There were significant correlation between occurrence of ischemic stroke and dyslipidemia. When the total cholesterol (Chol) increased by 1 mmol/L, the incidence of stroke will increase by 25% (Zhang et al., 2003; Huang and Zhang, 2011). In the systematic review, four trails mentioned the blood lipid change, including Chol and TG. The meta-analysis demonstrated that there were significant differences between the experimental and control groups in Chol and TG (*MD* = -1.06, 95% CI [-1.21, -0.92], *P* < 0.00001; *MD* = -1.05, 95% CI [-1.12, -0.97], *P* < 0.00001). Accordingly, blood lipids level may be proposed as one of the important indicators for the evaluation of efficacy of XNJ for treating cerebral infarction in the further clinical studies.

The efficacy and adverse events associated with XNJ in treatment of cerebral infarction were explored via extensive researches and strict methodologies. However, there are limitations to this research, such as the quality of the included data for the original trials. Many RCTs do not employ strict methodologies. Additionally, most of the involving patients were Chinese. It is necessary to including more varied population sample. The sample size, selection criteria varied for the included studies. We were unable to perform a subgroup analysis.

## Conclusion

These findings reflect that XNJ may significantly improve overall response rate, neural functional, Hemodynamics and ADL, and decrease the blood viscosity, serum levels of MMPs, the level of blood lipid and infarction size. It is likely that XNJ also decreases the Glasgow Coma Scale, but the results were not statistically significant. However, our findings must be interpreted with caution because of the small sample size and limitations of the study. Several rigorous, large-scale RCTs are necessary to confirm these results.

## Author Contributions

XM, YY, and TW performed the search and wrote the manuscript. NC and QX analyzed the data. YY, TW, and XH performed the data extraction. XM and JW designed the study and amended the paper.

## Conflict of Interest Statement

The authors declare that the research was conducted in the absence of any commercial or financial relationships that could be construed as a potential conflict of interest.
